# Fluorescence staining of live cyanobacterial cells suggest non-stringent chromosome segregation and absence of a connection between cytoplasmic and thylakoid membranes

**DOI:** 10.1186/1471-2121-8-39

**Published:** 2007-09-03

**Authors:** Dirk Schneider, Eva Fuhrmann, Ingeborg Scholz, Wolfgang R Hess, Peter L Graumann

**Affiliations:** 1 Institut für Biochemie und Molekularbiologie, Zentrum für Biochemie und Molekulare Zellforschung, Albert-Ludwigs-Universität Freiburg, Stefan-Meier-Strasse 19, 79104 Freiburg, Germany; 2 Institute for Experimental Bioinformatics Faculty for Biology, Universität Freiburg, Schänzlestr. 1, 79104 Freiburg, Germany; 3 Institute of Microbiology, Faculty for Biology, Universität Freiburg, Schänzlestr. 1, 79104 Freiburg, Germany

## Abstract

**Background:**

In spite of their abundance and importance, little is known about cyanobacterial cell biology and their cell cycle. During each cell cycle, chromosomes must be separated into future daughter cells, i.e. into both cell halves, which in many bacteria is achieved by an active machinery that operates during DNA replication. Many cyanobacteria contain multiple identical copies of the chromosome, but it is unknown how chromosomes are segregated into future daughter cells, and if an active or passive mechanism is operative. In addition to an outer and an inner cell membrane, cyanobacteria contain internal thylakoid membranes that carry the active photosynthetic machinery. It is unclear whether thylakoid membranes are invaginations of the inner cell membrane, or an independent membrane system.

**Results:**

We have used different fluorescent dyes to study the organization of chromosomes and of cell and thylakoid membranes in live cyanobacterial cells. FM1-43 stained the outer and inner cytoplasmic membranes but did not enter the interior of the cell. In contrast, thylakoid membranes in unicellular *Synechocystis *cells became visible through a membrane-permeable stain only. Furthermore, continuous supply of the fluorescent dye FM1-43 resulted in the formation of one to four intracellular fluorescent structures in *Synechocystis *cells, within occurred within 30 to 60 minutes, and may represent membrane vesicles. Using fluorescent DNA stains, we found that *Synechocystis *genomic DNA is compacted in the cell centre that is devoid of thylakoid membranes. Nucleoids segregated very late in the cell cycle, just before complete closing of the division septum. In striking contrast to *Bacillus subtilis*, which possesses an active chromosome segregation machinery, fluorescence intensity of stained nucleoids differed considerably between the two *Synechocystis *daughter cells soon after cell division.

**Conclusion:**

Our experiments strongly support the idea that the cytoplasmic and thylakoid membranes are not directly connected, but separate entities, in unicellular cyanobacteria. Our findings suggest that a transport system may exist between the cytoplasmic membrane and thylakoids, which could mediate the extension of thylakoid membranes and possibly also protein transport from the cytoplasmic membrane to thylakoid membranes. The cell cycle studies in *Synechocystis *sp. PCC 6803 show that the multiple chromosome copies per cell segregate very late in the cell cycle and in a much less stringent manner than in *B. subtilis *cells, indicating that chromosomes may become segregated randomly and in a passive fashion, possibly through constriction of the division septum.

## Background

The bacterial cell cycle is characterized through key events such as initiation of replication, termination of this process, segregation of chromosomes, and finally cell division. While replication is generally initiated through the DnaA protein, which recruits the replication apparatus to the origin regions of replication [1], division is instigated by the FtsZ protein, which forms a ring structure at the cell centre and subsequently recruits all further proteins required for formation of a division septum [2]. Bacteria that contain only a single chromosome, such as *Escherichia coli *and *Bacillus subtilis*, contain a machinery that actively separates duplicated chromosome regions soon after these have been replicated [3]. Thus, chromosome segregation occurs during ongoing replication, and is driven by an active mechanism. Both replication forks are stationary at the cell centre, and may contribute to the segregation mechanism through pushing of newly replicated DNA towards opposite cell poles. The SMC (MukB in *E. coli*) condensation complex forms subcellular centres within each cell half and is essential for the complete separation of chromosomes, possibly through locally condensing replicated DNA within each condensation centre [4]. Actin-like MreB proteins have also been implicated in active segregation of chromosomes [5]. However, so far it has been unclear how multiple chromosomes may be segregated in bacteria such as cyanobacteria. Cyanobacteria are a large phylum within the bacteria and perform oxygenic photosynthesis. Many cyanobacteria show circadian rhythms, and within these light/dark cycles, distinct cell-cycle dependent gene expression patterns have been described [6]. However, the circadian system operates independent from and even in the absence of proper cell division [7,8]. Very little is known about the normal cell cycle in cyanobacteria grown under constant light conditions. FtsZ is essential in *Synechocystis*, and appears to assemble in alternating division planes, same as the essential ZipN protein, which is unique to cyanobacteria and plants, and which interacts with FtsZ. The MinCDE system is required for the formation of proper FtsZ rings, but not essential for viability, and affects cell morphology, most likely through an effect on FtsZ ring positioning [9].

In addition to an outer and cytoplasmic membrane, cyanobacteria contain thylakoid membranes that harbour the membrane-bound complexes of the photosynthetic electron transfer chain. The initial steps of biogenesis of at least one photosystem have been suggested to take place in the cytoplasmic membrane. The pre-assembled core of this photosystem should subsequently be transferred to the thylakoid membranes, where it completely assembles [10]. However, so far it has not been clear if a direct connection exists between the two cyanobacterial inner membrane systems, although structural studies have suggested that the membranes are separate entities [11]. In this case, a protein transfer system must exist, which mediates the transfer of proteins and lipids from the cytoplasmic membrane to the thylakoids. The "vesicle inducing protein in plastids 1" (VIPP1), has been proposed to be involved in a putative vesicle transfer system in chloroplasts and cyanobacteria [12,13], but the connection between the two cyanobacterial inner membranes still remains unsolved.

Here we analyze the membranes and the cell cycle of the unicellular cyanobacterium *Synechocystis *sp. PCC 6803 by fluorescence microscopy. We have applied different fluorescent membrane stains to study the organization of the cytoplasmic and thylakoid membrane in living cells. The rational was to use a fluorescent dye (FM1-43) that does not cross the cell membrane, and a dye that is able to traverse membranes to analyse if a direct link exists between inner and thylakoid membranes. Our experiments suggest that thylakoid membranes are not directly connected to the cytoplasmic membrane in *Synechocystis*. However, continuous supply of FM1-43 resulted in formation of one to four intracellular vesicle-like structures, which indicates the existence of a possible active transport system between membranes. Visualization of *Synechocystis *genomic DNA showed that chromosomes are segregated very late in the cell cycle, just before complete closing of the septum, apparently through a random mechanism.

## Results

### Green membrane stains FM1-43 and mitotracker stain the outer and cytoplasmic membranes, or the thylakoid membranes, respectively, and show no direct link between cell and thylakoid membranes

**Figure 1:**
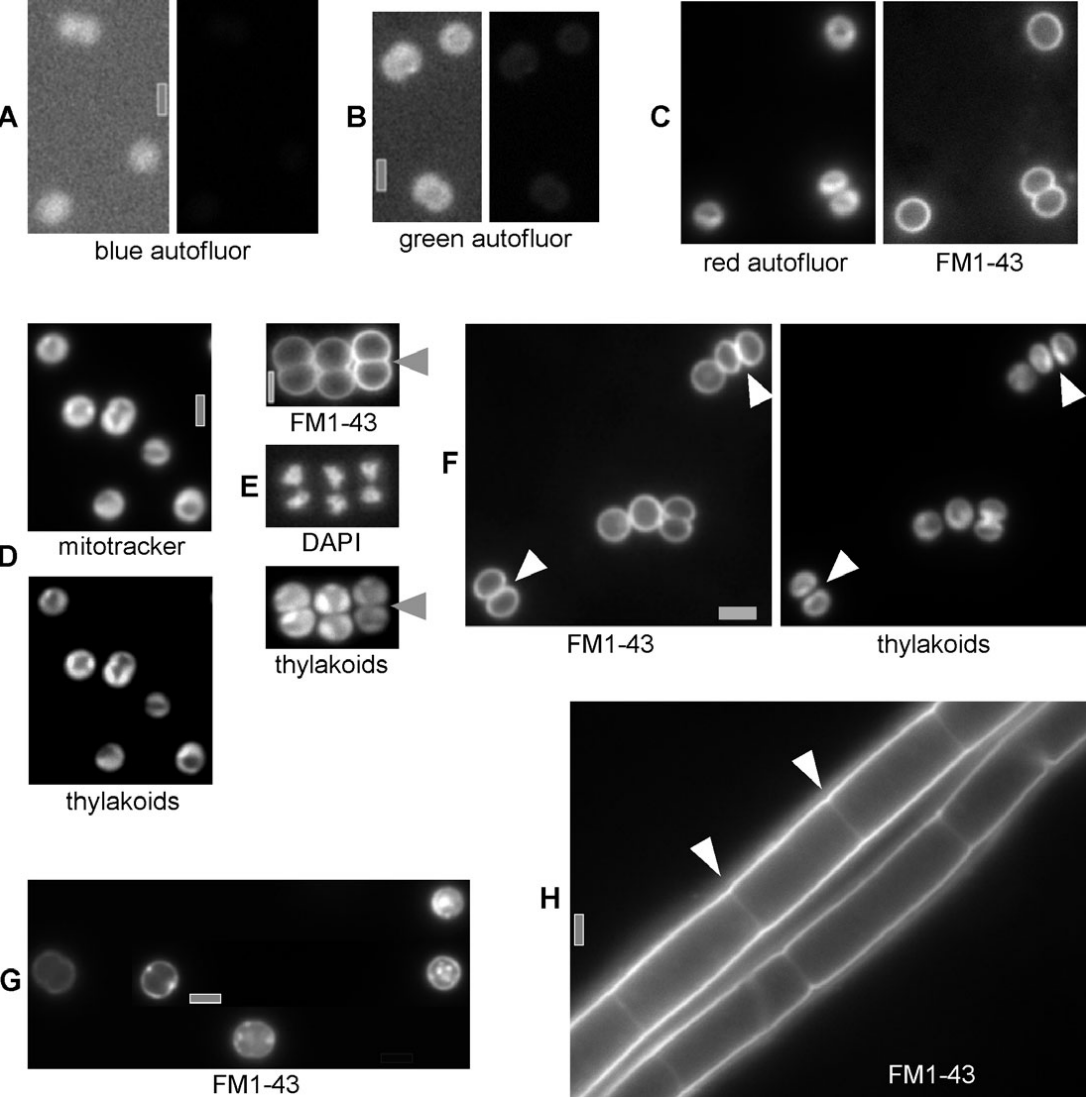
Fluorescence microscopy of exponentially grown *Synechocystis *cells. A) cells in the blue channel, right panel is scaled to the middle (blue) panel in E, B) cells in the green channel, right panel is scaled to the right (green) channel in C and to D, C) cells in the red channel (left panel, autofluorescence) and in the green channel after addition of FM1-43 membrane stain. D) cells in the green channel (upper panel) after addition of mitotracker membrane stain, or in the red channel (autofluorescence of proteins within thylacoids). E) FM1-43 membrane stain (upper panel), DAPI DNA stain (middle panel) and thylakoid autofluorescence (lower panel), grey triangles indicate division septa between separated thylakoids, F) FM1-43 stain and thylakoid autofluorescence, white triangles indicate outer membranes that have completely separated daughter cells, G) FM1-43 stain of cells subjected to conditions inducing plasmolysis (note that this is a composite image), H) *Fischerella *sp. cells stained with FM1-43 (green channel), white triangles indicate septa between cells. Grey bars 2 μm.

Due to the high degree of autofluorescence it is difficult to study subcellular structures in cyanobacteria by fluorescence microscopy. However, we found that there is very little fluorescence in the blue fluorescence channel (UV excitation/blue emission, see material and methods) (Fig. 1A, right panel is scaled like the DAPI panel in Fig. 1E), and in the green channel (blue excitation/green emission) (Fig. 1B, right panel is scaled like the FM1-43 panel in Fig. 1C and like Fig. 1D). Thus, *Synechocystis *genomic DNA can be efficiently stained with DAPI (Fig. 1E, middle panel), and the green membrane stains FM1-43 as well as mitotracker can be used in the green filter (Fig. 1C, right panel and 1D). FM1-43 is unable to cross the cell membrane in eukaryotic cells and in Gram-positive bacteria, while mitotracker green can traverse membranes, and thus stains mitochondria, or engulfed forespores in *Bacillus subtilis *cells [14]. Fig. 1C and 1D clearly show that FM1-43 and mitotracker green stain different parts of *Synechocystis *cells. While FM1-43 shows a uniform staining of the periphery of the cell (Fig. 1C, right panel), mitotracker stains the thylakoids, which have also strong autofluorescence in the red filter (green excitation/red emission) (Fig. 1C, left panel). To investigate if FM1-43 stains only the outer membrane, or both outer and cytoplasmic membranes in *Synechocystis*, several control experiments were performed. Firstly, cells were put to hyperosmotic conditions for 15 min. Because only the inner membrane shows plasmolysis bays in Gram negative bacteria, the presence of membrane-proximal fluorescent patches in Fig. 1G clearly shows that FM1-43 also stains the inner membrane. Secondly, inner membranes could clearly be seen as septa in the filamentous cyanobacterium *Fischerella *sp. (Fig. 1H). Along the lateral side of filaments, outer and inner membranes are seen as bright single membrane (they are spaced less than 250 nm apart and thus appear as single line), whereas between cells, only a weak membrane staining is visible, corresponding to the inner membrane that partitions single cells (Fig. 1H). Thirdly, in some *Synechocystis *cells, the thylakoid membranes have already separated between daughter cells, indicating the presence of a septum between daughter cells, while the outer membrane has not yet completely separated both cells (compare cells in Fig. 1F, indicated by white triangles, where the membrane signal between daughter cells is about twice as strong as on the outer surface of the cells, with cells in Fig. 1E). In rare cases, separation of thylakoids occurs when the outer membranes have not deeply invaginated between daughter cells (Fig. 1E), and the cell membrane between daughter cells is weakly but clearly visible, while staining of the thylakoids is similar to background fluorescence (compare Fig. 1E, upper and lower panels). Because proteins diffuse through membranes on a time scale of few μm per second (0.13 μm^2 ^s^-1^) [15], diffusion of a membrane stain is extremely fast and cannot account for the differential staining of cell and thylakoid membranes. It was, however, possible that the FM1-43 fluorescence was not observed in thylakoid membranes due to an efficient quenching of the dye fluorescence by thylakoid membrane attached phycobilisomes. To exclude this possibility, we have isolated membranes from *Synechocystis *cells under high salt conditions, under which a large portion of the phycobilisomes remain attached to the thylakoid membranes. A fraction of these membranes was extensively washed with a low salt buffer with 0.5% of the detergent Triton X-100. With this buffer the phycobilisomes were almost completely removed from the thylakoid membranes, as proven by absorbance spectroscopy. When the dye FM1-43 was added to these membranes and the fluorescence emission of the membrane solution was measured in an Aminco Bowman Series II fluorimeter after excitation of the dye at 470 nm, we did not observe any differences in fluorescence emission and thus no quenching of the FM1-43 fluorescence by phycobilisomes (data not shown). Therefore, FM1-43 and mitotracker can distinguish between the cytoplasmic and intracellular membrane compartments, and these observations suggest that there is no direct link between cytoplasmic and thylakoid membranes, or else a weak staining of the thylakoids would be expected after addition of FM1-43.

### Continuous labelling with FM1-43 visualizes intracellular bodies

**Figure 2:**
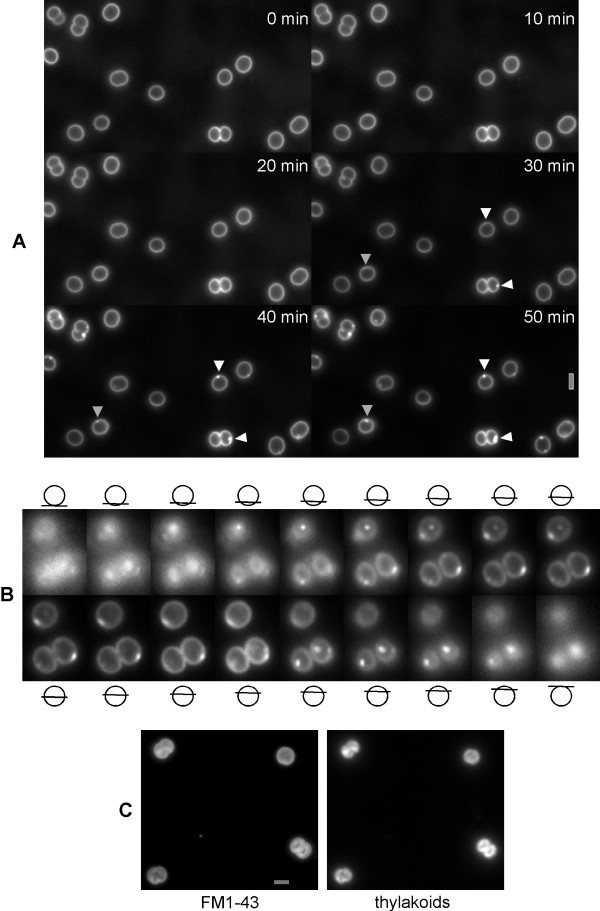
Continuous addition of FM1-43 to growing *Synechocystis *cells. A) Time course of cells incubated on FM1-43 containing agarose, white triangles indicate arising patches of FM1-43, grey triangle a moving FM1-43 patch. B) Z-series through cells after incubation on FM1-43 containing agarose for 45 min, position within the focal plane is indicated above/below the images. Grey bars 2 μm.

The absence of a direct connection between the cytoplasmic membrane and thylakoids in *Synechocystis *suggests that parts of the cytoplasmic membrane may be invaginated and fused to thylakoid membranes for their continued synthesis during cell growth or for transfer of proteins between the two membranes. To test this idea, we added FM1-43 to the agarose pads containing growth medium, onto which cells were added to fix them in one plane. Continued supply of FM1-43 resulted in the formation of 1 to 4 (on average 2.3, with 65 cells analysed) fluorescent patches or dots close to the cytoplasmic membrane (Fig. 2A). In very rare cases, 5 patches were observed. These structures were only seen 20 min after addition of cells to the slides, and on average only after 30 min (Fig. 2A). The structures persisted for at least 2 hours, and did not move much within the cells, although different patterns between 10 min intervals were observed in 8 out of 55 cells analysed (see grey triangle in Fig. 2A). Fluorescence intensity of cells with continuous supply of FM1-43 increased over time, for at least 2 hours, and FM1-43 signals became apparent within thylakoid membranes in many (but not all) cells (Fig. 2C). However, care must be taken with the interpretation of the latter finding, as we have no means to investigate if the cells are still in their normal physiological state after 2 hours, although there is no strong argument against their well-being.

To obtain more information about the nature of the observed fluorescent bodies close to the cytoplasmic membrane, we obtained Z-series through the cells. Fig. 2B shows a representative 200 nm increment series through two cells. In the single round cell, two fluorescent patches are apparent, one below and one around the central plane. In the larger, almost fully divided cell, at least 4 patches can be seen, two below and two above the central plane. In general, larger cells contained more fluorescent patches than smaller cells, in agreement with their larger volume.

### Cells remain in a round morphology for a large fraction of the cell cycle, and nucleoids separate completely just before completion of cell division

**Figure 3:**
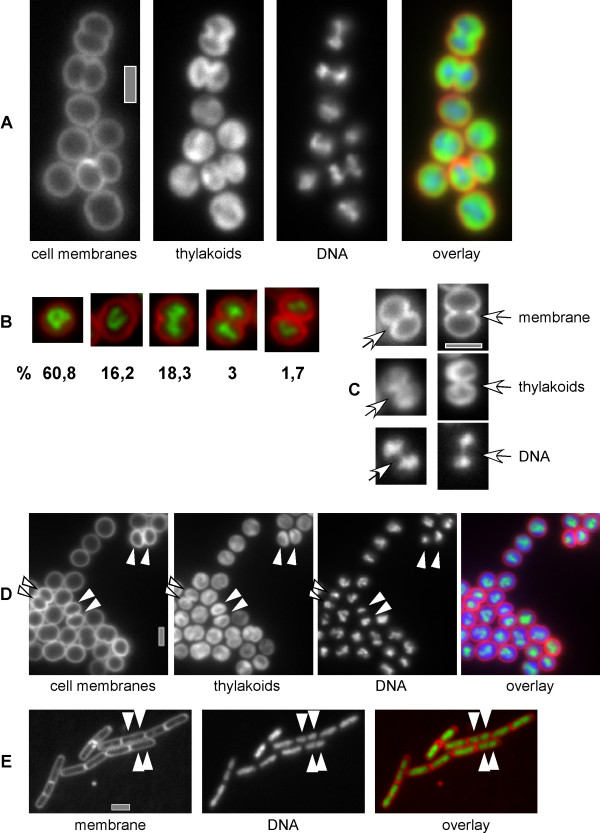
Cell cycle of *Synechocystis*. A) Growing cells stained with DAPI (DNA) and FM1-43 (membranes), overlay of membranes (red), thylakoids (green) and DNA (blue). B) Overlay of membrane (red) and DNA (green) of representative cells of the 5 classes defined in the text. Numbers indicate the percentage of cells present in a certain stage in an exponentially growing culture C) Fine structure of DNA (DAPI) structure in cells with a deep membrane (FM1-43) constriction and non-separated thylakoids (autofluorescence). Arrows point out non-separated nucleoids at the division plane. D) Exponentially growing cells stained with FM1-43 (membrane), DAPI (DNA), white triangles point out completely separated nucleoids having considerably different staining intensity, boxed triangles point out roughly equal nucleoids. E) Exponentially growing *Bacillus subtilis *cells, membrane and DNA stain analogous to panels A-D, white triangles indicate separated nucleoids in cells that do not yet have a visible division septum. Grey bars 2 μm.

Labelling of cells with the fluorescent dyes DAPI or Hoechst 333 showed that the DNA occupies central intracellular spaces devoid of thylakoid membranes (in 92% of all cells, with >250 cells analysed), whereas the thylakoid membranes occupy the periphery of the cells (Fig. 3A). In the remaining 8% of the cells, DNA stain was present directly adjacent to the cell membranes. Usually, a single (occasionally irregularly shaped) nucleoid was present in round cells (Fig. 3A and first 2 panels of 2B), and a bilobed nucleoid in dividing cells (Fig. 3B, 3^rd ^and 4^th ^panel, Fig. 3C). Infrequently, single round cells contained 2 up to 4 distinct DAPI-stained regions (7.5% of all cells analysed), and were thus more complex than the majority of the cells. The morphologies of thylakoid membranes were highly heterogenic, thylakoid membranes occupied the cell periphery at every possible pattern (Fig. 3A–D).

We wished to obtain a quantitative view on the cell cycle in *Synechocystis*. To this end, cell morphologies were grouped into 5 different patterns, according to the progression through the cell cycle. Group 1 comprised entirely round cells, which must be early in the cell cycle. Group 2 was defined as cells that are elongated, and thus clearly growing but have no visible membrane constriction (because elongated cells can look round when they are vertical to the plane of the view, this group may be slightly underrepresented). Group 3 comprises cells that have a clear membrane invagination, and group 4 those cells with deep invagiations that are not yet closed. Cells in which outer membranes have completely surrounded both daughter cells, but which are still connected, were scored as group 5. Figure 3B shows representatives of each group, and reveals that most cells in an exponentially growing culture belong to group 1. 16% of the cells were clearly elongated, and thus initiating to divide, while 18% had clear membrane invaginations at the division plane, and were thus actively dividing. 3% of the cells had deep invaginations, while less than 2% were fully divided but still connected (with 437 cells analysed). These data indicate that *Synechocystis *cells grow and gradually divide, to quickly separate once outer membranes have fully separated daughter cells.

Only 1 out of 80 cells from group 3 had completely separated thylakoids, and none had 2 completely separated nucleoids. About 50% of the cells of group 4 had separated thylakoids and exhibited two distinct nucleoids, while all cells from group 5 had separated thylakoids and 2 nucleoids. All cells from group 4 that still contained non-separated thylakoids (i.e. 50%, Fig. 3C, middle panel) showed nucleoids that were still connected by thin DNA threads. These observations show that nucleoids are separated very late in the cell cycle, just before the division septum closes (as seen by separated versus non-separated thylakoids). This is in contrast to *E. coli *and *B. subtilis *cells, in which nucleoids are generally well separated before the septum closes [3]. Fig. 3E shows an example of two *B. subtilis *cells in which nucleoids are completely separated well before a division septum is detectable.

### Chromosome segregation in *Synechocystis *is less stringently controlled than in *B. subtilis*

**Figure 4:**
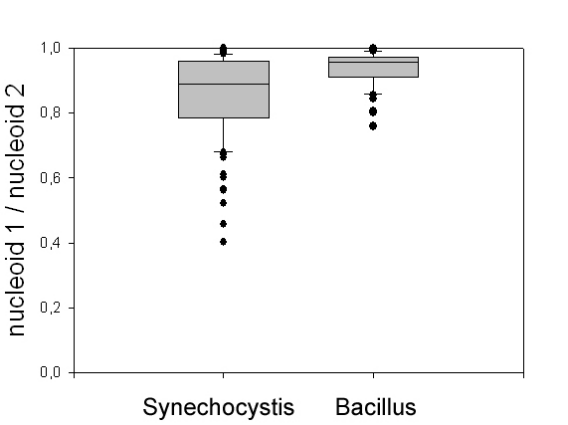
Distribution of the relative DNA content of daughter cells of dividing *Synechocystis *cells based on DAPI stains. Of two daughter cells the smaller DNA content was each divided by the larger one, the ratios are represented by the grey bars. In *Bacillus *the relative nucleoid ratios varied between 0.76–1 with a mean of 0.94 and a standard deviation of 0.05. In *Synechocystis *the relative ratio scattered from 0.4–1 with a mean of 0.85 and a standard deviation of 0.13.

Having several copies of the chromosome, *Synechocystis *cells may not require an elaborate chromosome segregation machinery as is operative in many bacteria that contain only a single chromosome (or two before cell division). DAPI can be used to quantify the amount of DNA in a cell; therefore, we sought to investigate if fluorescence intensity of DAPI can shed light on the question if chromosome segregation occurs in a tightly regulated fashion in *Synechocystis*. Because nucleoids occupy only the central portion of *Synechocystis *cells (while the outer parts are occupied by the thylakoid membranes, see above), their width (or diameter, which is equal to their depth because the cells are round) is comparable to the width and depth of nucleoids in *B. subtilis *cells. On average, *Synechocystis *nucleoids were 1.12 μm wide, while *B. subtilis *nucleoids were 0.96 μm wide, showing that there is no considerable difference in focal depth between nucleoids of the two organisms. Size and intensity of completely separated nucleoids (or those connected by a thin thread, see above) was frequently strikingly different between *Synechocystis *daughter cells (Fig. 3D, indicated by white triangles), in contrast to cells of *B. subtilis *(Fig. 3E). Intensities of separated nucleoids were measured in more than 100 cells using Metamorph 6 software. In contrast to *B. subtilis *cells, in which the scatter of staining intensity was relatively low, fluorescence intensity of *Synechocystis *nucleoids varied to a much higher degree (Fig. 4). In *B. subtilis*, the relative ratios of nucleoids per daughter cells varied between 0.76–1 with a mean of 0.94 and a standard deviation of 0.05, while in *Synechocystis *daughter cells, the relative ratio scattered from 0.4–1 with a mean of 0.85 and a large standard deviation of 0.13. These results prove that chromosome segregation occurs less stringently in *Synechocystis *than in *B. subtilis*, and suggest that possibly, segregation operates by a passive mechanism. This idea is supported by our finding that nucleoids are only completely separated when thylakoid membranes are also separated by the septum.

## Discussion

In the present report, we have studied the cell cycle of the unicellular cyanobacterium *Synechocystis *sp. PCC 6803 by fluorescence microscopy. Under the chosen growth conditions, *Synechocystis *cells obtain a more oval shape instead of a round shape in the second half of the cell cycle, and show a visible constriction in the last quarter of the cell cycle, to quickly separate after complete division. Interestingly, transcription of many genes is cell cycle dependent in cyanobacteria, and transcription of rRNA and of genes encoding ribosomal proteins mainly occurs at the first part of the cell cycle [16]. However, these data correspond to cells grown under alternating dark/light cycles, and not under constant light as used in this study.

Similar to the well studied model-organisms *E. coli *and *B. subtilis*, inhibition of DNA replication in the cyanobacterium *Microcystis aeruginosa *results in a complete block of cell division [17], showing that cell division and DNA replication are tightly coupled and controlled. Several genes encoding protein involved in cell division have been identified in *Synechocystis *[18], and a chromosome partition protein SMC homolog, which is essential for the complete separation of chromosomes in *E. coli *and *B. subtilis *[19,20], is present in *Synechocystis *{gene *sll1120*, [21]}. While actin-like MreB proteins are involved in chromosome segregation in *B. subtilis*, *E. coli *and *C. crescentus *cells [22-24], an MreB homolog is not encoded in *Synechocystis*, although homologs are present in other, rod-shaped cyanobacteria. Therefore, MreB may control cell morphology in cyanobacteria rather than being critical for chromosome segregation. While DNA replication appears to be coupled to cell division in cyanobacteria [17], essentially nothing is known about how cyanobacteria like *Synechocystis *separate chromosomes into the daughter cells. The finding that cyanobacteria such as *Synechocystis *contain 10–20 identical copies of the chromosome [25] could permit absence of an active chromosome segregation machinery, or allow for a non-stringent segregation machinery. Our observation that in *Synechocystis *the chromosomes are distributed considerably more randomly to daughter cells than in *B. subtilis *provides evidence for a relaxed control or absent active machinery for chromosome segregation. In support of this view, we have found that complete nucleoid separation does not precede cell division, in contrast to other bacteria like *e.g. E. coli *and *B. subtilis *cells, but that nucleoids are separated very late during the *Synechocystis *cell cycle. The nucleoids possibly segregate passively through the closing of the division septum, based on the finding that nucleoids were almost never completely separated in cells having a constriction at the division plane but non-separated thylakoid membranes (Fig. 3). In contrast, chromosomes segregate normally in the absence of cell division in *E. coli *and *B. subtilis *[26]. Thus, chromosome segregation appears to occur through a mechanism largely different from the known active systems. A tight connection between nucleoid partitioning and cell division has also been observed in the cyanobacterium *Synechococcus *sp. PCC 9308 [27], and seems therefore to be common to several cyanobacteria. In toto, these data suggest that chromosomes may be separated randomly during the cell cycle, resulting in populations with different chromosome copy numbers, in which daughter cells replicate the chromosomes following separation to regain a normal average copy number. While this report was under review, Hu *et al*. have shown that chromosomes also separate randomly in the filamentous cyanobacterium *Anabaena *sp. PCC 7120, even in the absence of MreB [28], which strongly supports the model of random passive chromosome segregation in cyanobacteria.

Besides analyzing the *Synechocystis *cell cycle, we have also investigated the distribution of the fluorescent dye FM1-43, which selectively stains the outer membrane as well as the cytoplasmic membrane in *Synechocystis *(Fig. 1). While in *Synechocystis*, as well as in *Fischerella *sp., the outer and cytoplasmic membranes were clearly stained with FM1-43, we did not observe staining of thylakoid membranes. This observation suggests that there is no direct connection between the two inner membrane systems in *Synechocystis *and in *Fischerella*. It is still under debate whether in cyanobacteria the two inner membrane systems are connected or not. In a recent study based on electron microscopy it has been suggested that the two membranes are not connected [11], although in comprehensive studies it has been argued that not observing a connection does not necessarily mean that the membranes are not connected [29,30]. The finding that FM1-43 does significantly stain the *Synechocystis *cytoplasmic membrane, but not the thylakoids, supports the view that the two membranes are not connected. Interestingly, after continuous application of FM1-43 we observed the formation of fluorescent bodies within the cells (Fig. 2), indicating that the fluorescent dye may become transferred to other intracellular structures on a longer time scale. While we have not yet identified the exact nature of these structures, it is possible that the dye has been transferred to lipid bodies via the cytoplasmic membrane. Cyanobacterial cells contain lipid bodies, which have been found in close connection to the cytoplasmic membrane [29]. It is, however, also possible that the observed fluorescent bodies represent membrane vesicles. A vesicle transfer between the cyanobacterial membranes has been proposed in the recent years [12], and a recent report has provided powerful support of such structures. In line with our observations and suggestions, Nevo *et al*. have observed intracellular vesicles in the cyanobacterium *Microcoleus *sp., and these vesicles were either isolated from or attached to membranes [31].

## Conclusion

We have found strong supportive evidence for two possibly distinct features of cyanobacterial cells: absence of stringent segregation of chromosomes, suggesting that cyanobacterial multicopy chromosomes may be segregated at random by a passive mechanism, and lack of a direct connection between cell and thylakoid membranes, indicating that these membrane systems are separate entities. Based on our time course experiments, it is tempting to speculate that vesicles and/or lipid bodies serve to transport proteins and/or lipids, between the two internal membrane systems. However, it will be necessary to unravel the exact nature of the observed fluorescent bodies and to further analyse membrane dynamics and of membrane proteins by fluorescent microscopy.

## Methods

### Growth conditions

A glucose tolerant *Synechocystis *sp. PCC 6803 wild type strain was grown photomixotrophically in liquid BG11 medium [32] supplemented with 10 mM glucose at 34°C under 40 μE/m^2^s of cold white light. Cells were harvested in the mid-log growth phase for subsequent analyses. *Fischerella *sp. were grown in liquid BG11 without glucose at 28°C under 40 μE/m^2^s white light. For induction of plasmolysis, an exponentially growing cell culture was diluted 1:1 with a 1 M sucrose solution, yielding 0.5% sucrose final concentration.

### Fluorescent dyes, microscopy, and image acquisition

Exponentially growing cells were mounted on 1% agarose pads containing growth medium for microscopy. FM1-43FX and Mitotracker green (Molecular Probes, Netherlands) were added to cells at 2.5 μg/ml or 5 μg/ml, respectively, DAPI (blue DNA-specific membrane permeable stain) was added at 200 ng/ml, and FM1-43FX was added at 2.5 μg/ml to agarose pads for continued supply of membrane stain. The red variant of FM1-43FX, FM4-64, stains the cell membrane of the Gram positive bacterium *Bacillus subtilis*, but not fully engulfed forespore, whereas Mitotracker green stains the membrane of fully engulfed spores [14], as well as mitochondria in eukaryotic cells, which are not stained by FM1-43FX. Thus, Mitotracker green is a membrane permeant stain, while FM1-43FX can not cross (inner) cell membranes. Images were acquired on an Olympus AX70 upright microscopy using a 100× objective (A = 1.45), a MicroMax CCD camera, and Metamorph 6.0 software (Universal Imaging, USA). For acquisition of Z-stacks, 20 planes with a spacing of 0.2 μm were taken from bottom to top, or reverse. Signal intensities were measured in Metamorph program. Filter specificities were as follows: "blue" ex360-370, dc400, em420-460, "green" ex460-495, dc505, em510-550, "red" ex480-550, dc570, em590. Usually, 100 ms exposure times were used.

### Membrane preparation and fluorescence spectroscopy

1 l of *Synechocystis *cells were grown to an OD_700 _of about 1. After centrifugation at 3000 rpm the cells were resuspended in 2 ml 750 mM K-Phosphate, 150 mM Tricine (pH 7.2) buffer and the cell were subsequently broken with a Mini BeadBeater (Biospec Products Inc.). Unbroken cells and cellular debris was removed by centrifugation at 3000 rpm (4°C), and the supernatant was further separated into membranes and soluble proteins by an ultracentrifugation step (40000 rpm, 30 min, 4°C). The sedimented membranes were either resuspended in high salt buffer (as above) or in a low salt buffer (50 mM Na-Phosphate, pH 7.2) to which 300 mM glycerol and 0.5% Triton X-100 were added. The solubilized membranes were sedimented again as described above. This washing step was repeated three times and the membranes were afterwards washed two times with low salt buffer in the absence of glycerol and detergent. By these washing steps most of the membrane attached phycobilisomes were removed. After determination of the chlorophyll concentrations of phycobilisome containing and phycobilisome-less membranes, UV/Vis spectra were recorded on a Perkin Elmer Lambda 35 UV/Vis spectrometer to proof removal of the phycobilisomes. To 1 ml membranes with a chlorophyll concentration of 10 μg/ml 1 μl FM1-43 was added and fluorescence emission spectra were recorded in the range between 480 – 750 nm after excitation of the dye at 470 nm. Fluorescence spectra were recorder at room temperature with an Aminco Bowman Series II fluorescence spectrometer.

## Competing interests

The author(s) declare that they have no competing interests.

## Authors' contributions

D.S. and P.L.G. performed all microscopical experiments, designed the study and wrote the manuscript. E.F. performed control experiments with isolated *Synechocystis *membranes. I.S. provided cells of *Fischerella *and helped with microscopy. W.R.H. helped design and write the manuscript.
